# Mercerized mesoporous date pit activated carbon—A novel adsorbent to sequester potentially toxic divalent heavy metals from water

**DOI:** 10.1371/journal.pone.0184493

**Published:** 2017-09-14

**Authors:** Abdullah Aldawsari, Moonis Ali Khan, B. H. Hameed, Ayoub Abdullah Alqadami, Masoom Raza Siddiqui, Zeid Abdullah Alothman, A. Yacine Badjah Hadj Ahmed

**Affiliations:** 1 Department of Chemistry, College of Science, King Saud University, Riyadh, Saudi Arabia; 2 School of Chemical Engineering, Engineering Campus, Universiti Sains Malaysia, Nibong Tebal, Penang, Malaysia; Institute of Materials Science, GERMANY

## Abstract

A substantive approach converting waste date pits to mercerized mesoporous date pit activated carbon (DPAC) and utilizing it in the removal of Cd(II), Cu(II), Pb(II), and Zn(II) was reported. In general, rapid heavy metals adsorption kinetics for *C*_*o*_ range: 25–100 mg/L was observed, accomplishing 77–97% adsorption within 15 min, finally, attaining equilibrium in 360 min. Linear and non-linear isotherm studies revealed Langmuir model applicability for Cd(II) and Pb(II) adsorption, while Freundlich model was fitted to Zn(II) and Cu(II) adsorption. Maximum monolayer adsorption capacities (*q*_*m*_) for Cd(II), Pb(II), Cu(II), and Zn(II) obtained by non-linear isotherm model at 298 K were 212.1, 133.5, 194.4, and 111 mg/g, respectively. Kinetics modeling parameters showed the applicability of pseudo-second-order model. The activation energy (*E*_*a*_) magnitude revealed physical nature of adsorption. Maximum elution of Cu(II) (81.6%), Zn(II) (70.1%), Pb(II) (96%), and Cd(II) (78.2%) were observed with 0.1 M HCl. Thermogravimetric analysis of DPAC showed a total weight loss (in two-stages) of 28.3%. Infra-red spectral analysis showed the presence of carboxyl and hydroxyl groups over DPAC surface. The peaks at 820, 825, 845 and 885 cm^-1^ attributed to Zn–O, Pb–O, Cd–O, and Cu–O appeared on heavy metals saturated DPAC, confirmed their binding on DPAC during the adsorption.

## Introduction

Industrial discharges are the major carrier of heavy metals to surface and sub-surface water, limiting clean water availability for human usage, consequently posing potential health hazards [1, 2]. The tendency to bio-accumulate, non-degradability, and persistency are the some of the major attributes of heavy metals. Among heavy metals, cadmium [Cd(II)] and lead [Pb(II)] belongs to a class of highly toxic (carcinogenic or suspected carcinogen) divalent heavy metals present in aqueous systems, while copper[Cu(II)] and zinc [Zn(II)] in trace amounts are essential for the normal growth and development of human beings. But in high amounts, Cu(II) can cause gastrointestinal catarrh, hemochromatosis, and skin dermatitis brasschills [3], while Zn(II) can cause metal fume fever and restlessness [4]. Due to their detrimental consequences on human health strict regulation have been imposed by various countries for limiting their levels in waste effluents and in drinking water supplies. The maximum allowable limits set by World Health Organization (WHO) for Cd(II), Pb(II), Zn(II), and Cu(II) in drinking water are 0.003,0.010, 3.0, and 2.0 mg/L, respectively [5]. According to Saudi Arabian Standards Organization’s (SASO) the respective maximum allowable limits for aforementioned heavy metals in bottled drinking water are 0.003, 0.010, 0.1, and 1 mg/L [6]. Therefore, to conserve human health and sustain natural water reservoirs quality it is essential to check and minimize heavy metals in waste effluents before discharging them to water reservoirs and in drinking water supplies.

Chemical precipitation, biodegradation, electrocoagulation, flocculation, and oxidation are some of the conventional water treatment techniques. The diversified nature of the pollutants present in waste effluents restricts these techniques in water quality rectification as per treatment standards. Adsorption is considered as an apotheosis treatment process on account of its non-specificity towards the pollutants irrespective of their diverse nature [7]. Both carbonaceous [8, 9] and non-carbonaceous [3, 10] adsorbents have been used in the removal of heavy metals from aqueous medium. But on economic and ecological grounds, the carbonaceous adsorbents derived from plant and animal waste precursors have an upper hand over non-carbonaceous adsorbents and commercially available activated carbons. Moreover, these carbonaceous adsorbents are highly porous with large internal surface area [11].

Date palm tree is a native of Middle East Asian and North African region of the world. It produces date fruit, which is a major food crop of these regions. Saudi Arabia is one of the largest worldwide producers of date fruit. Statistical data revealed that over 157,000 hectares of land with 25 million palm trees produces 1.1 million tons of dates annually. It is reported that Saudi Arabia exports only 5% of their date fruit, rest are consumed locally [12]. Morphologically, date fruit consist of edible fleshy pericarp covering hard endocarp (seed) commonly called date pit (DP), which is 6–12% of date fruit [13]. Chemically, DP is composed of moisture: 5–10%, ash: 1–2%, protein: 5–7%, oil: 7–10%, crude fiber: 10–20%, and carbohydrates: 55–65% [14]. In common practice, DP is thrown after the consumption of edible date fruit pulp and is considered as a waste. Research is going on to explore the substantive utilization methods of waste DP. Some researchers have focused on exploring the medicinal applications [15], while others have worked to explore the nutritional values [16] of DP. Environmental scientists are not far behind in digging out DP applications for environmental conservation.

Date plant wastes, because of it low cost and significant adsorption potential in the removal of wide range of environmental pollutants has gained considerable attention as a precursor material for the preparation of carbonaceous adsorbents by physical and chemical activations [17]. The physically (steam) and chemically (phosphoric acid, H_3_PO_4_; potassium hydroxide, KOH; nitric acid, HNO_3_) activated DP carbon (AC) was developed and utilized for the adsorption of Pb(II) and Cd(II) from water. The observed maximum adsorption capacities for Pb(II) and Cd(II) were 139 and 129 mg/g, respectively [18]. The adsorption behavior of Cu(II) and Cd(II) on chemically (sodium hydroxide, NaOH) activated AC from DP was explored with maximum adsorption capacities for Cu(II) and Cd(II) were 118.1 and 88.4 mg/g, respectively [19]. Al-Ghouti et al [20] have utilized raw DP in the removal of Cu(II) and Cd(II). Slow adsorption kinetics for both Cu(II) and Cd(II) reaching equilibrium after 72 hr with maximum adsorption capacities for Cu(II) and Cd(II) as high as 35.9 and 39.5 mg/g, respectively, were observed. Oubekka and co-workers have synthesized TEMPO/NaBr/NaOCl modified DP and tested its adsorptive potential in removal of Cd(II) and Pb(II). The observed maximum adsorption capacities for Cd(II) and Pb(II) were 8.6 and 55.6 mg/g, respectively [21]. Calcium oxide activated DP carbon was prepared and tested in the removal of Cu(II) and Ni(II) from water [22]. The maximum adsorption capacities observed for Cu(II) and Ni(II) at 293 K were 37.3 and 29.9 mg/g, respectively, while the equilibration time was 180 min. The adsorption of Pb(II) and Zn(II) was tested on sulphuric acid activated DP carbon [23]. The maximum removal of Pb(II) (19.6 mg/g) and Zn(II) (10.6 mg/g) was observed at pH: 6.0 and 293 K, while the observed equilibration time was 60 min. Though, there are many studies reporting the utilization of raw and modified DP for sequestering heavy metals from aqueous phase. But to the best of our knowledge upon extensive literature survey most of the studies have reported the utilization of raw and modified DP for the removal of one or two heavy metals from water.

Previously, we have developed DP char, chemically activate it with alkaline metal hydroxides viz. NaOH and KOH, and optimize its physical and chemical activation conditions to obtain highest yield of DP activated carbon (DPAC) and maximum methylene blue adsorption capacity [24]. Herein, a comprehensive study focusing the utilization of previously optimized DPAC for the adsorptive removal of four potentially toxic divalent heavy metals viz. Cd(II), Pb(II), Cu(II), and Zn(II) from aqueous medium was carried out. DPAC was well characterized to understand it surface chemistry before and after heavy metals adsorption. To test the economic feasibility of synthesized DPAC desorption experiments were carried out.

## Materials and methods

### Chemical and reagents

1000 mg/L stock solutions of Cd(II), Pb(II), Cu(II), and Zn(II) were prepared by using their nitrate salts purchased from BDH Chemical, England. Hydrochloric acid (HCl) was purchased from Sigma-Aldrich, Germany. Sodium hydroxide (NaOH) was procured from Merck, Germany. The other chemicals and reagents used were of analytical reagent (A.R) grade or as specified. Deionized (D.I) water was used throughout the experiments.

### Preparation of DPAC

The DPs were collected, washed with D.I water, dried overnight in a vacuum oven at 80°C to remove moisture. The dried DPs were mechanically ground and sieved to 250 μm particle size. The DPs powder was carbonized in a furnace at 500°C for 2 hr at a heating rate of 20°C/min. The carbonized DPs (DPC) powder was activated with NaOH. The DPC powder and NaOH were mixed in 1:3 (wt.: wt. %) ratio. 10 mL of D.I water/gram of NaOH was added to make a suspension. The suspension was continuously stirred over a magnetic stirrer for 6 hr. Thereafter, the suspension was dried in an oven at 100°C for 24 hr. Furthermore, it was transferred to a boat shaped crucible and was placed in a tubular furnace, heated to 800°C for 1.5 hr under 100 mL/min N_2_ flow. After 1.5 hr, it was cooled to room temperature inside a tubular furnace. The prepared DPAC was washed several times with 0.1M HCl until it attained pH: ~ 1, the DPAC was further washed with hot D.I water until it achieved pH: ~ 6.5. The DPAC was separated by using cellulose membrane filter of size 0.45 μm. and dried at 110°C for 24 hr in an oven.

### Characterization of DPAC

Fourier-transform infrared (FT-IR) spectrometer (FT-IR; Nicolet 6700 FTIR Thermo Scientific) was employed to depict the functional groups present on DPAC surface (before heavy metals adsorption) and to find out the functional groups actively participating in heavy metals binding over DPAC surface (after heavy metals adsorption). The thermal stability of DPAC was tested by thermo gravimetric (TGA; Mettler Toledo TGA/SDTA851 with Starc software) analyzer in temperature range: 25–850°C, under N_2_ flow at a heating rate of 10°C/min. The surface morphology and elemental composition of DPAC before and after heavy metals adsorption was determined by scanning electron microscope (SEM; Hitachi Co., Japan, Model No. S3400N) coupled with energy dispersive X-ray (EDX; Thermo Scientific Co., USA).

### Batch adsorption studies

100 mL Erlenmeyer flask containing 0.05 g of DPAC was equilibrated with 50 mL heavy metal solution of initial concentration (*C*_*o*_): 50 mg/L on isothermal water bath shaker at 100 rpm and 298 K. At equilibrium, the sample was filtered by Whatman filter paper No 41 and residual heavy metal concentration was determined using atomic absorption spectrometer (Perkin Elmer: Pin AAcle 900T). The effect of heavy metal solution pH on adsorption was studied for initial pH (pH_i_) range: 1–9. The contact time study was carried out for range: 1–1440 min at varied heavy metal *C*_*o*_ range: 25–100 mg/L and temperatures (T): 298 K. Isotherm and thermodynamic studies were also carried out for *C*_*o*_ range: 25–300 mg/L and T range: 298–318 K. The heavy metals adsorption capacities at equilibrium and at time t and % adsorption were determined as:

$$q_{e}\left(mg/g\right)=\left(C_{o}-C_{e}\right)\times \frac{V}{m}$$ (1)

$$q_{t}\left(mg/g\right)=\left(C_{o}-C_{t}\right)\times \frac{V}{m}$$ (2)

$$\%\;adsorption=\frac{C_{o}-C_{e}}{C_{o}}\times 100$$ (3)

where *V* is the volume of solution (L), *m* is the mass of DPAC(g), *C*_*o*_, *C*_*e*_ and *C*_*t*_ are the initial, equilibrium, and at time *t* concentrations of heavy metals in aqueous solutions, respectively.

### Desorption studies

To evaluate the economic feasibility of the synthesized DPAC, desorption studies were carried out in batch mode. Initially, DPAC (0.05 g) was saturated with 50 mL heavy metal solution of *C*_*o*_: 50 mg/L for 1440 min. At equilibrium, solid phase (adsorbent) was separated and was washed several times with D.I. water to remove heavy metal ions traces. To elute adsorbed heavy metals ions, saturated adsorbent was treated with 50 mL of HCl (0.1 and 0.05 M), HNO_3_ (0.1 and 0.05 M), and H_2_SO_4_ (0.1 and 0.05 M), CH_3_COOH (0.1 and 0.05 M) for 1440 min on water bath shaker under ambient temperature (298 K) conditions. The desorbed heavy metal ions concentration was quantitatively analyzed and % desorption was calculated as:

$$\%desorption=\frac{Concentration\;of\;heavy\;metal\;ions\;desorbed\;by\;eluent}{Initial\;concentration\;of\;heavy\;metal\;ions\;adsorbed\;on\;DPAC}\times 100$$ (4)

To ensure repeatability during the study, both batch mode adsorption and desorption experiments were carried out twice and only average values have been reported.

## Results and discussion

### Adsorption studies

Preliminary experiments were carried out to test the adsorption potential of DPAC for eight potentially toxic heavy metals in single-metal system. **S1 Fig** illustrates the order of heavy metals adsorption on DPAC. Among the tested heavy metals, least (2.22 mg/g) adsorption was observed for Cr(VI), followed by Cr(III) < Ni(II) < Co(II) < Zn(II) < Pb(II) < Cu(II) < Cd(II). The adsorption of Cd(II) on DPAC was highest (44.9 mg/g) among the tested heavy metals. Therefore, Cd(II), Zn(II), Pb(II), and Cu(II) having comparatively better adsorption performance among the tested heavy metals, and having the adsorption order: Cd(II) > Cu(II) > Zn(II) > Pb(II) were selected for detailed adsorption studies.

In general, the adsorption of heavy metal is governed by their hydrated radii, hydration energies, electronegativity, and heavy metal aqueous phase solubility [25]. The hydrated radii, hydration energies, and electronegativity of Cd(II), Zn(II), Pb(II), and Cu(II) are 4.26, 4.30, 4.01, and 4.19 Å; -1807,-1955, -1481, and -2010 kJ/mol; and 1.69, 1.65, 2.33, and 2.00 Pauling, respectively. Smaller the hydrated radius of heavy metal, faster and quantitatively larger is its adsorption over carbon surface. The hydration energy permits the detachment of water molecules from heavy metal cation in aqueous phase, and thereby reflects the ease with which the heavy metal cation interacts with carbon surface during the adsorption. Therefore, more a heavy metal cation hydrated, stronger is its hydration energy, and lesser it can interact with the adsorbent [26]. Additionally, higher the electronegativity of heavy metal, larger will be its tendency to quantitatively adsorb [27]. Considering aforementioned interpretations, the adsorption of studied heavy metals on DPAC should follow different orders. The order of heavy metals adsorption on DPAC should be Pb(II) > Cu(II) > Cd(II) > Zn(II) if hydrated radii and electronegativity are considered. If hydrated energies are considered, the order should be Pb(II) > Cd(II) > Zn(II) > Cu(II). Contrarily, the experimentally observed heavy metals adsorption order was different during the study, confirming that the adsorption not only depends on the heavy metal ions properties, but also on the physical (viz. surface area, pore size) and chemical (viz. functional groups and surface charge) properties of DPAC.

**Fig 1:**
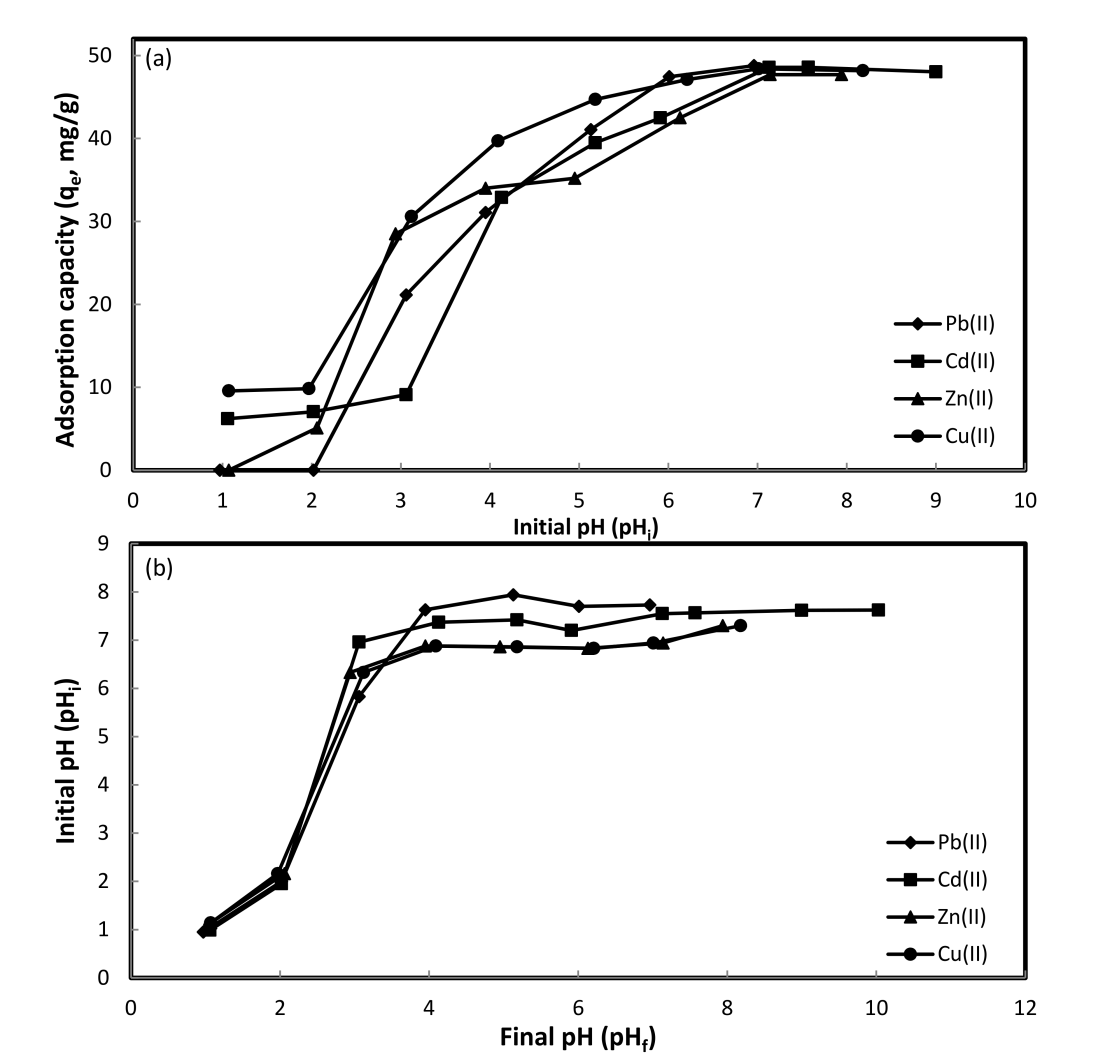
Effect of pH on heavy metals adsorption **(a)**, Plot of initial pH (before) and final pH (after) heavy metals adsorption **(b)** onto DPAC (**Experimental conditions:** m: 0.05 g, t: 24 hr, *C*_*o*_: 50 mg/L, V– 50 mL, agitation speed– 100 rpm, T– 298 K).

**Fig 1A** presents the adsorption of Pb(II), Cd(II), Zn(II), and Cu(II) on DPAC for an initial pH (pH_i_) range: 1–9. The adsorption of Cd(II), Pb(II), Cu(II), and Zn(II) for studied pH_i_ range and *C*_*o*_: 50 mg/L increased from 6.2 to 48.6 mg/g; 0 to 48.4 mg/g; 9.6 to 48.4 mg/g; and 0 to 48.2 mg/g, respectively. At lower pH_i_ values, excessive protonation of DPAC surface rendered heavy metals binding, resulting lower adsorption on DPAC. As pH_i_ values increases, a competition between protons and heavy metal ions to occupy DPAC surface sites decreases, thereby increasing heavy metal ions adsorption on DPAC. Additionally, after the accomplishment of heavy metals adsorption under highly acidic conditions, an increase in final pH (pH_f_) values towards neutral pH values was observed (**Fig 1B**), suggesting that neutralization and adsorption were parallel processes [28]. The pH_f_ values remains more or less neutral upon further increase in pH_i_ values during the study. At higher pH_i_ values, precipitation of heavy metals due to the formation of metal oxides occurred. The reported pH_i_ values for Cd(II), Pb(II), Zn(II), and Cu(II) precipitation were 9.2 [29], > 7 [27], >8 [9], and 8.5 [30] Thus, pH_i_: 7 was optimized for the adsorption studies.

**Fig 2:**
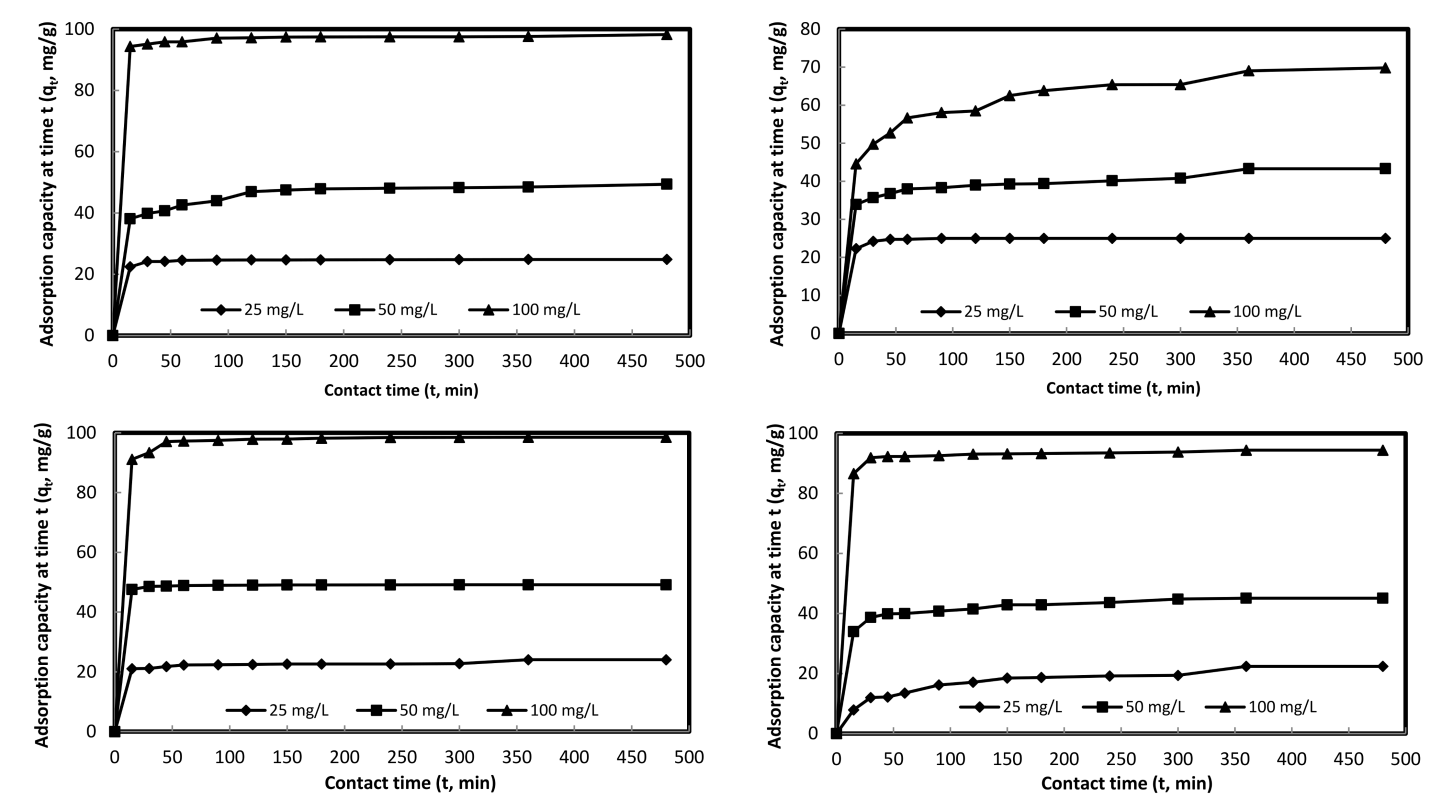
Effect of contact time on Cd(II) (a), Zn(II) (b), Cu(II) (c), and Pb(II) (d) adsorption at varied concentrations onto DPAC. (**Experimental conditions:** m: 0.05 g, V: 50 mL, agitation speed: 100 rpm, T: 298 K, pH_i_: 7).

**Fig 2A–2D** illustrates the contact time plots for Cd(II), Zn(II), Cu(II), and Pb(II) adsorption at varied initial concentrations (*C*_*o*_) viz., 25, 50, and 100 mg/L on DPAC. Overall, the initial heavy metals adsorption at varied *C*_*o*_ was rapid, as depicted by a steep slopes, accomplishing 77–96%, 63.8–97.3%, 87.5–96.8%, 35–91.7% adsorption of Cd(II), Zn(II), Cu(II), and Pb(II), respectively within 15 min of contact time, finally, attaining equilibrium in 360 min, as depicted by a plateau region of the plots. But slower adsorption kinetics were observed for Zn(II) at *C*_*o*_: 100 mg/L and for Pb(II) at *C*_*o*_: 25 mg/L, accomplishing 63.8 and 35% adsorption in 15 min, respectively. Additionally, for initial 15 min, there was a proportional decrease in Zn(II) and increase in Pb(II) adsorption with an increase in *C*_*o*_, while irregular adsorption trends were observed for Cd(II) and Cu(II) under similar experimental conditions. The equilibrium adsorption capacities of Cd(II), Zn(II), Cu(II), and Pb(II) for studied *C*_*o*_ range varied between 24.8 and 98.2 mg/g; 25 and 69.8 mg/g; 24.1 and 98.5 mg/g; and 22.4 and 94.4 mg/g, respectively. Similar equilibration times (360 min) were observed for Cd(II) adsorption onto bamboo charcoal at *C*_*o*_: 100 mg/L with 18.20 mg/g as an equilibrium adsorption capacity [31] and for Zn(II) adsorption on CMOC at *C*_*o*_: 50 mg/L with 40 mg/g as an equilibrium adsorption capacity [9]. Comparatively faster equilibration time (180 min) for Pb(II), Cu(II), and Hg(II) adsorption onto AC derived from waste coconut buttons with *C*_*o*_ range: 25–100 mg/L was reported previously, while the magnitude of their respective equilibrium adsorption capacities were 12.45–44.40 mg/g; 11.92–36.40 mg/g; and 12.21–40.77 mg/g [32], which were comparatively less. Kula et al [33] observed 90 min as an equilibration time for Cd(II) adsorption on AC derived from olive stone with 1.65 mg/g as an equilibrium adsorption capacity at *C*_*o*_: 45 mg/L and T: 30°C. Slower kinetics (1440 min) was observed for Cu(II) and Zn(II) adsorption onto CS600 biochar having 10.2 and 7.8 mg/g as their respective equilibrium adsorption capacities [34]. The observed equilibration time was 240 min for Cd(II) adsorption on PAC with 15.7 mg/g as an equilibrium adsorption capacity at *C*_*o*_: 100 mg/L and T: 30°C [35].

### Adsorption modeling

#### Adsorption isotherms

**Fig 3:**
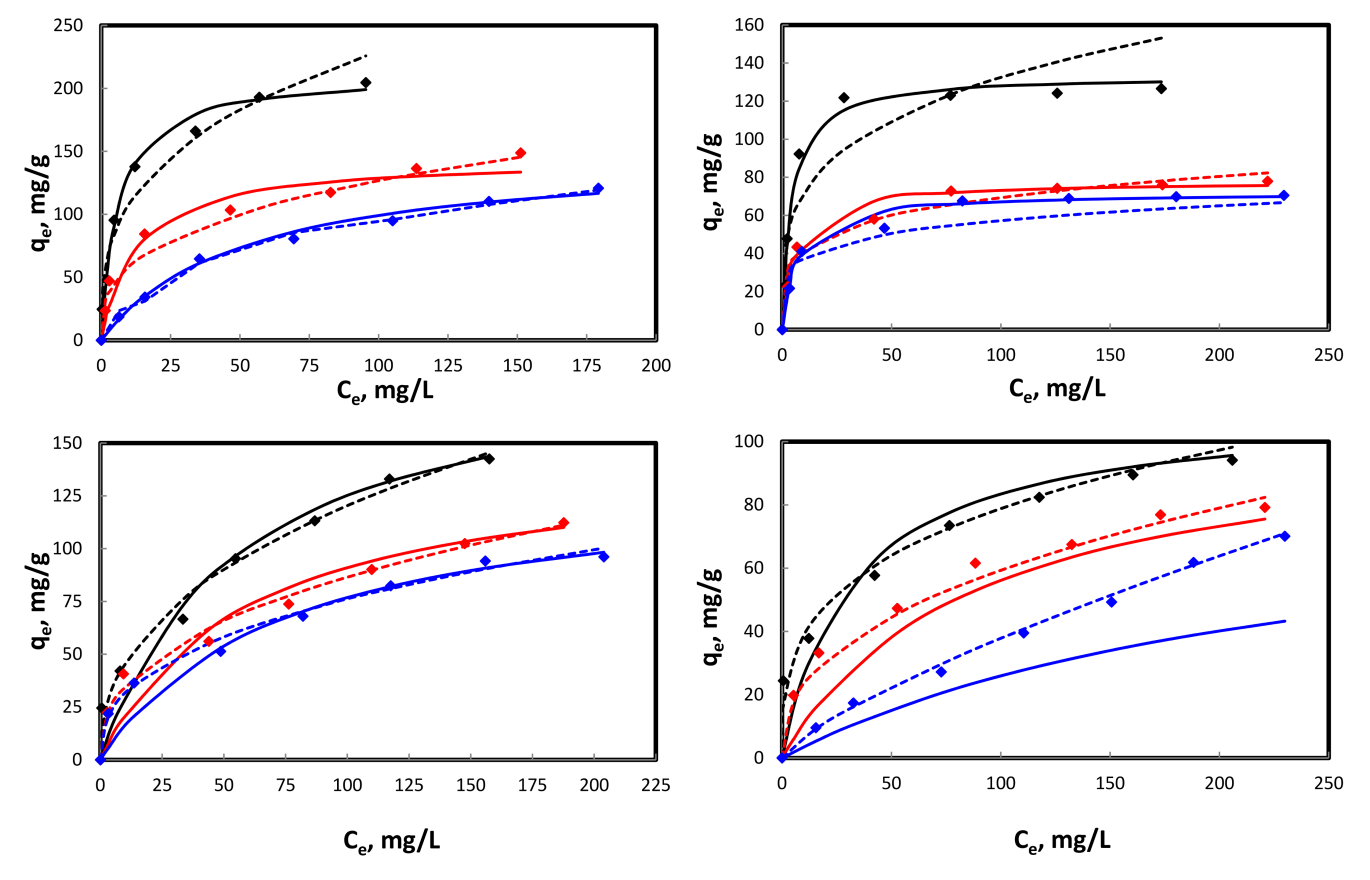
Non-linear isotherms for Cd(II) **(a)**, Pb(II) **(b)**, Cu(II) **(c)**, and Zn(II) **(d)** adsorption on DPAC [♦: q_e,exp._; ___: Langmuir isotherm; ….: Freundlich isotherm at 25 (black), 35 (red), and 45 (blue)°C].

The two parameters Langmuir [36] and Freundlich [37] isotherm models (both in linear and non-linear forms) were applied to adsorption data at varied temperatures viz. 25, 35, 45°C. Detailed information regarding models is given in Supplementary Information (**S1 Text**). **Tables 1 and 2** presents linear and non-linear isotherms data. The magnitudes of maximum monolayer adsorption capacities (*q*_*m*_) for Pb(II), Cd(II), Cu(II), and Zn(II) at 25°C depicted by non-linear isotherm model were 133.49, 212.10, 194.45, and 111 mg/g, respectively, decreases with increase in temperature. Similar *q*_*m*_ trend with different magnitudes was observed through linear isotherm model. The regression coefficient (*R*^*2*^) values for the adsorption of Pb(II) and Cd(II) on DPAC (obtained from linear and non-linear models) showed the applicability of Langmuir model, well supported by non-linear isotherm plots (**Fig 3A and 3B**), while Freundlich model was better fitted to Cu(II) and Zn(II) adsorption data, further confirmed by their non-linear isotherm plots (**Fig 3C and 3D**). The separation factor (*R*_*L*_) values for heavy metals adsorption on DPAC at varied temperatures were well in range of favorable adsorption process. The degree of adsorption represented by Freundlich constants *K*_*f*_, decreased with increase in temperature showing less significant adsorption at higher temperature. The magnitudes of *n* for studied heavy metals at varied temperatures were well in range of physical adsorption process.

**Table 1:** Linear isotherm data for heavy metals adsorption on DPAC.

Temperature (K)	Linear isotherm models						
	Langmuir				Freundlich		
	*q*_*m*_ (mg/g)	*b* (L/mg)	R_L_	R^2^	*K*_*f*_ (mg/g)(L/mg)^1/n^	*n*	R^2^
**Pb(II)**							
298	129.9	0.2391	0.0137	0.9993	33.2	3.33	0.7748
308	80.0	0.1120	0.0289	0.9982	21.9	3.96	0.9177
318	73.5	0.1027	0.0314	0.9982	19.1	3.84	0.9125
**Cd(II)**							
298	217.4	0.1488	0.0219	0.9805	42.2	2.62	0.9406
308	149.2	0.0668	0.0475	0.9947	26.7	2.96	0.9268
318	128.2	0.0635	0.0499	0.9968	16.4	2.66	0.9381
**Cu(II)**							
298	156.25	0.0407	0.0757	0.9444	16.68	2.34	0.9836
308	121.95	0.0325	0.0930	0.9487	16.12	2.76	0.9822/
318	107.52	0.0276	0.1077	0.9217	13.95	2.80	0.9692
**Zn(II)**							
298	100	0.0532	0.0590	0.9819	20.18	3.39	0.8016
308	84.0	0.0424	0.0729	0.9710	14.62	3.20	0.9853
318	79.4	0.1850	0.0177	0.9382	6.18	2.35	0.9666

**Table 2:** Non-linear isotherm data for heavy metals adsorption on DPAC.

Temperature (K)	Non-linear isotherm models						
	Langmuir				Freundlich		
	*q*_*m*_ (mg/g)	*b* (L/mg)	R_L_	R^2^	*K*_*f*_ (mg/g)(L/mg)^1/n^	*n*	R^2^
**Pb(II)**							
298	133.49	0.2233	0.0147	0.9703	38.84	3.76	0.8091
308	77.93	0.1524	0.0214	0.9813	25.71	4.64	0.9662
328	72.14	0.1327	0.0245	0.9954	24.48	5.42	0.9759
**Cd(II)**							
298	212.10	0.1605	0.0203	0.9852	50.27	3.03	0.9490
308	144.62	0.0799	0.0400	0.9424	26.81	2.97	0.9411
328	150.23	0.0194	0.1466	0.9890	14.22	2.44	0.9553
**Cu(II)**							
298	194.45	0.0180	0.2358	0.9516	17.42	2.39	0.9821
308	144.85	0.0169	0.1647	0.9261	13.74	2.54	0.9844
328	134.43	0.0133	0.2004	0.9349	12.76	2.58	0.9483
**Zn(II)**							
298	111	0.0303	0.0991	0.9228	19.36	3.28	0.9798
308	105.69	0.0113	0.2278	0.8782	18.80	2.41	0/9785
328	88.96	0.0041	0.4484	0.5920	1.15	1.32	0.9716

### Adsorption kinetics

Kinetics data for the adsorption of Cd(II), Pb(II), Cu(II), and Zn(II) on DPAC was modeled by pseudo-first-order [38] and pseudo-second-order [39] kinetics models. Detailed information regarding models is given in Supplementary Information (**S2 Text**). **Table 3** presents kinetic parameters for the adsorption of Cd(II), Zn(II), Cu(II), and Pb(II) on DPAC at *C*_*o*_: 25–100 mg/L. Comparatively higher *R*^*2*^ magnitudes of pseudo-second-order kinetics model for Cd(II), Pb(II), Cu(II), and Zn(II) adsorption on DPAC within studied *C*_*o*_ range revealed the applicability of pseudo-second-order model. Additionally, the applicability of pseudo-second-order kinetics model was also confirmed by nearer *q*_*e*,*exp*._ and *q*_*e*,*cal*_ values for heavy metals adsorption on DPAC. During the study, a decrease in pseudo-second-order rate constant (*k*_*2*_) and an increase in heavy metals adsorption capacity with increase in *C*_*o*_ was observed. An increase in adsorption capacity was due an increase in driving force exerted by a greater pressure gradient at higher concentration. Furthermore, the *k*_*2*_ for heavy metals at *C*_*o*_: 25 mg/L was found to decrease in order: Zn(II) > Cd(II) > Cu(II) > Pb(II). At *C*_*o*_: 50 and 100 mg/L, the *k*_*2*_ values follows the order: Cd(II) > Cu(II) > Zn(II) > Pb(II) and Cu(II) > Cd(II) > Zn(II) > Pb(II), respectively indicating the favorability of different heavy metals at varied *C*_*o*_ to be adsorbed easily and rapidly on DPAC.

**Table 3:** Kinetics data for heavy metals adsorption on DPAC.

*C*_*o*_ (mg/L)	*q*_e,exp._ (mg/g)	Kinetics models					
		Pseudo-first-order			Pseudo-second-order		
		*q*_*e*,*cal*._ (mg/g)	*K*_*1*_ (1/min)	R^2^	*q*_*e*,*cal*_ (mg/g)	*K*_*2*_ (g/mg-min)	R^2^
**Cd(II)**							
25	24.83	1.02	0.0124	0.8595	24.87	0.0344	1
50	49.38	3.19	0.0051	0.7560	49.50	0.0130	0.9995
100	98.50	10.89	0.0090	0.9001	98.04	0.0077	1
**Zn(II)**							
25	24.99	4.79	0.0055	0.8862	25	0.0902	1
50	43.30	7.94	0.0041	0.9169	43.67	0.0099	0.9997
100	69.80	26.18	0.0001	0.9053	70.42	0.0010	0.9998
**Cu(II)**							
25	24.09	2.55	0.0028	0.7146	22.88	0.0310	1
50	49.15	1.03	0.0163	0.9195	49.26	0.0114	1
100	98.54	5.41	0.0163	0.9419	99.01	0.0085	1
**Pb(II)**							
25	22.40	11.95	0.0055	0.8848	23.81	0.0084	0.9923
50	45.10	10.54	0.0101	0.9218	45.87	0.0062	0.9996
100	94.40	3.66	0.0064	0.7721	94.34	0.0009	1

### Adsorption thermodynamics

The thermodynamics parameters viz. Gibb’s free energy change (ΔG°), standard enthalpy change (ΔH°) standard entropy change (ΔS°), and activation energy (*E*_*a*_) for the adsorption of Cd(II), Pb(II), Cu(II) and Zn(II) on DPAC were calculated.

Van’t Hoff’s equation was used to evaluate the magnitude of ΔS° and ΔH°, expressed as:

$$lnK_{c}=\frac{\Delta S{}^{\circ}}{R}-\frac{\Delta H{}^{\circ}}{R}\times \frac{1}{T}$$ (5)

$$K_{c}=\frac{C_{Ae}}{C_{e}}$$ (6)

where *R* (8.314 J/mol-K) is a universal gas constant, T (K) is the absolute temperature, *K*_*c*_ is the standard thermodynamics equilibrium constant, *C*_*Ae*_ (mg/L) and *C*_*e*_ (mg/L) are the equilibrium concentrations of heavy metals on solid and solution phases, respectively.

The magnitude of ΔG° was calculated as:

$$\Delta G{}^{\circ}=-RT\;lnK_{c}$$ (7)

The Dubinin–Radushkevich (D-R) isotherm model [40] was applied to calculate the magnitude of *E*_*a*_ for heavy metals adsorption on DPAC, mathematically expressed as:

$$lnq_{e}=lnq_{m}-\beta \varepsilon^{2}$$ (8)

where *q*_*m*_ (mmol/g) is the D-R monolayer capacity, *β* (mol^2^/kJ) is a constant related to adsorption energy, *ɛ* is Polanyi potential related to equilibrium concentration (*C*_*e*_).

$$\varepsilon =RT\;ln\left(1+\frac{1}{C_{e}}\right)$$ (9)

where *R* is the universal gas constant (8.314 J/mol-K), *T* (*K*) is the absolute temperature.

The *E*_*a*_ (kJ/mol) is calculated as:

$$E_{a}=\frac{1}{\sqrt{-2\beta \;}}$$ (10)

**Table 4** presents thermodynamics parameters for the adsorption of Zn(II), Cd(II), Cu(II), and Pb(II) on DPAC at *C*_*o*_: 25, 100 and 300 mg/L. The heavy metals adsorption at varied concentrations on DPAC was exothermic, as depicted by negative ΔH° values, agreeing well with previously carried out studies on dyes adsorption on different industrial wastes [41]. The ΔS° values for the adsorption of studied heavy metals were negative signifying a decrease in randomness at the solid/solution interface. Both negative and positive ΔG° values were observed indicating both spontaneous and non-spontaneous nature of heavy metals adsorption on DPAC. At lower heavy metals concentrations and temperature the ΔG° values were positive, but at higher concentration and temperature negative values were observed. The *E*_*a*_ values for the adsorption of heavy metals on DPAC were well in range of physical adsorption i.e. < 8 kJ/mol.

**Table 4:** Thermodynamics data for heavy metals adsorption on DPAC.

*C*_*o*_ (mg/L)	Thermodynamics parameters							
	E_a_ (kJ/mol)			ΔH° (kJ/mol)	ΔS° (J/mol-K)	ΔG° (kJ/mol)		
	298 K	308 K	318 K			298 K	308 K	318 K
**Zn(II)**								
25				-31.13	-82.34	6.61	5.72	4.97
100	0.707	0.707	0.500	-102.43	-324.51	6.14	1.59	-0.29
300				-34.20	-117.72	-0.78	-2.25	-3.12
**Cd(II)**								
25				-96.93	-290.12	10.68	7.14	4.91
100	2.236	0.745	0.408	-95.89	-296.82	7.52	4.32	1.59
300				-49.51	-159.61	2.12	-0.04	-1.04
**Cu(II)**								
25				-76.63	-227.09	9.54	5.44	5.08
100	1.581	0.500	0.500	-25.69	-76.76	2.86	1.95	1.33
300				-2.99	-17.64	-2.25	-2.47	-2.60
**Pb(II)**								
25				-120.49	-373.95	9.23	4.91	1.78
100	1.581	0.500	0.224	-58.43	-194.12	0.57	-1.33	-3.32
300				-15.97	-60.19	-1.94	-2.62	-3.14

### Desorption studies

**Fig 4:**
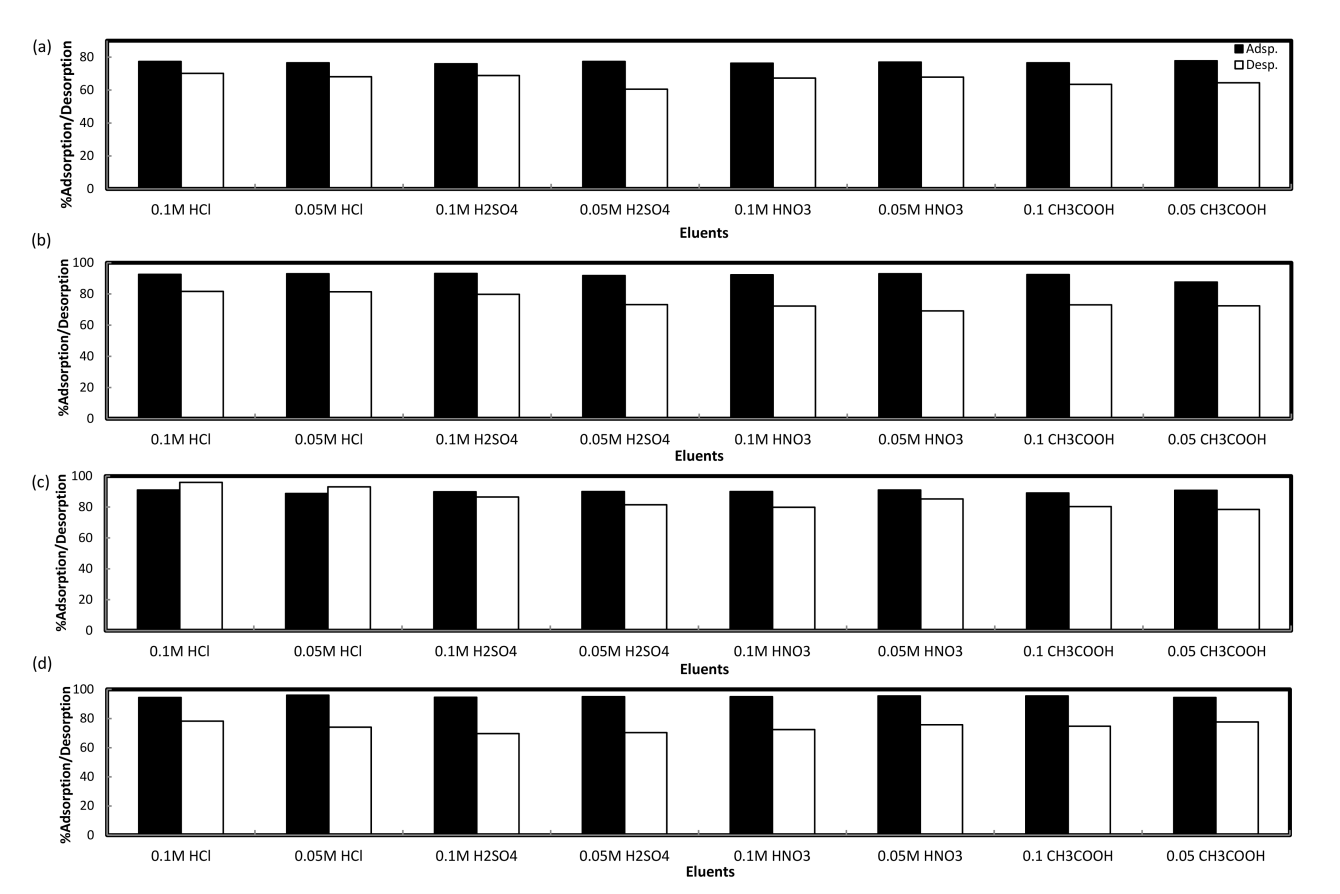
Desorption plots of Zn(II) (a), Cu(II) (b), Pb(II) (c), and Cd(II) (d) from DPAC by different eluents.

Cd(II), Zn(II), Pb(II), and Cu(II) ions were eluted from saturated DPAC during desorption studies as illustrated in **Fig 4**. HCl, H_2_SO_4_, HNO_3_, and CH_3_COOH solutions each of concentrations 0.05 and 0.1 M were tested as an eluents. The elution of Zn(II) was maximum (70.1%) with 0.1 M HCl followed by 0.1 M H_2_SO_4_ (68.9%) > 0.05 M HCl (68.1%) > 0.05 M HNO_3_ (67.9%) > 0.1 M HNO_3_ (67.3%) > 0.05 M CH_3_COOH (64.4%) > 0.1 M CH_3_COOH (63.5%) > 0.05 M H_2_SO_4_ (60.6%). The maximum amount (81.6%) of Cu(II) was eluted with 0.1 M HCl followed by 0.05 M HCl (81.4%) > 0.1 M H_2_SO_4_ (79.8%) > 0.05 M H_2_SO_4_ (73.2%) > 0.1 M CH_3_COOH (73%) > 0.05 M CH_3_COOH (72.4%) > 0.1 M HNO_3_ (72.2%) > 0.05 M HNO_3_ (69.1%). For Pb(II), maximum amount (96%) was eluted with 0.1 M HCl followed by 0.05 M HCl (93%) > 0.1 M H_2_SO_4_ (86.4%) > 0.05 M HNO_3_ (85.2%) > 0.05 M H_2_SO_4_ (81.5%) > 0.1 M CH_3_COOH (80.3%) > 0.1 M HNO_3_ (79.8%) > 0.05 M CH_3_COOH (78.4%). The elution of Cd(II) was maximum with 0.1 M HCl (78.2%) followed by 0.05 M CH_3_COOH (77.6%), 0.1 M CH_3_COOH (74.7%), 0.05 M HCl (74.1%), 0.05 M HNO_3_ (72.4%), 0.1 M HNO_3_ (70.3%), 0.05 M H_2_SO_4_ (70.3%), 0.1 M H_2_SO_4_ (69.7%). The maximum elution of studied heavy metals with strong acid 0.1 M HCl having pH: ~1 indicates the occurrence of ion-exchange process between divalent heavy metal ions and protons. During heavy metals elution process, the excessive numbers of protons from 0.1 M HCl form a shield around DPAC surface disrupting coordinating sphere of chelated heavy metal ions as they cannot compete with the protons resulting in their elution. Additionally, comparatively lower heavy metals elution with other strong acids viz., HNO_3_ and H_2_SO_4_ was possibly due to comparatively bigger ionic size of NO_3_^-^ and SO_4_^2-^ ions than Cl^-^ ion.

### Characterization of DPAC

**Fig 5:**
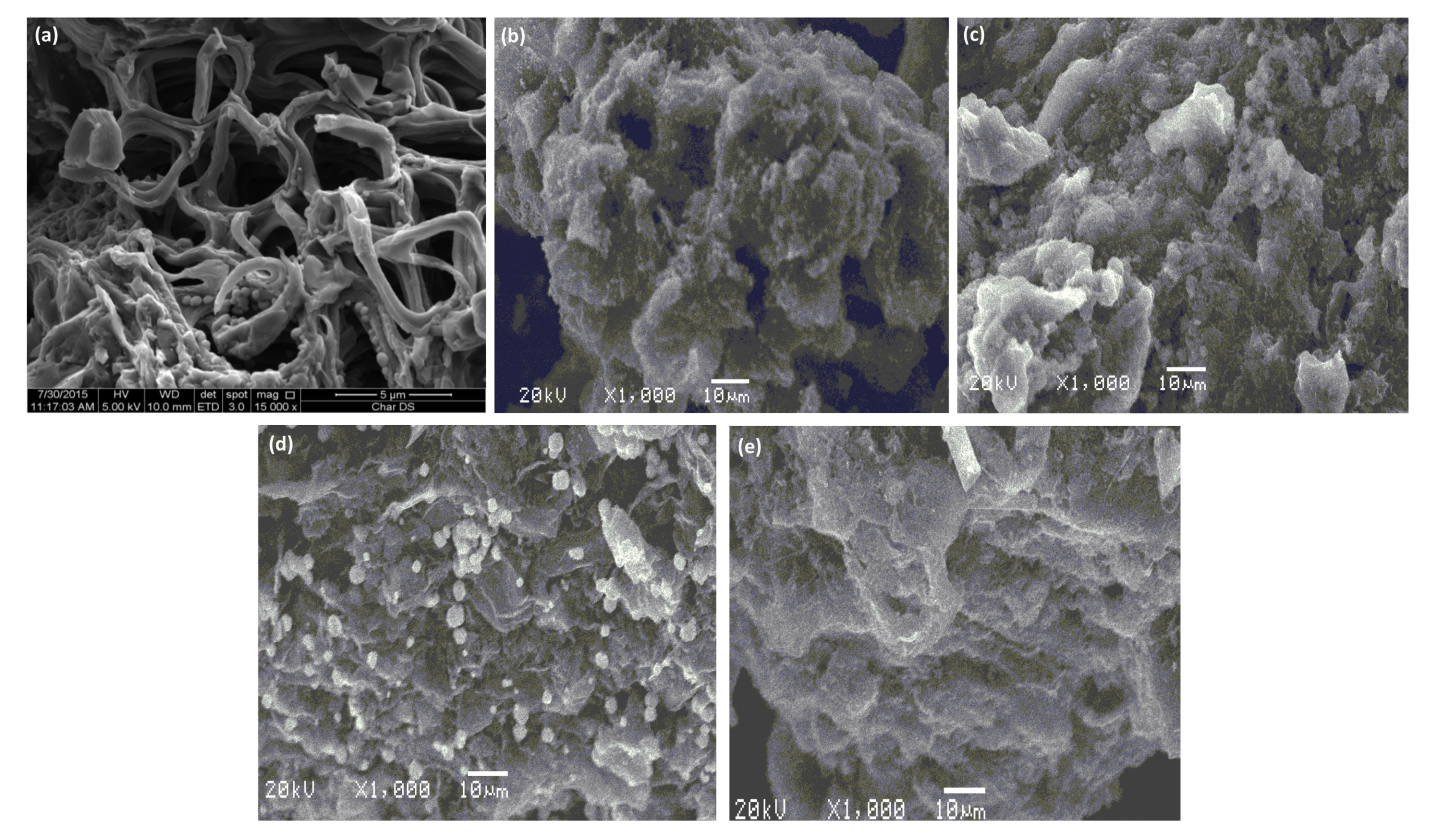
SEM images of DPAC before (a), and after Cu(II) (b), Zn(II) (c), Cd(II) (d), Pb(II) adsorption.

**Fig 6:**
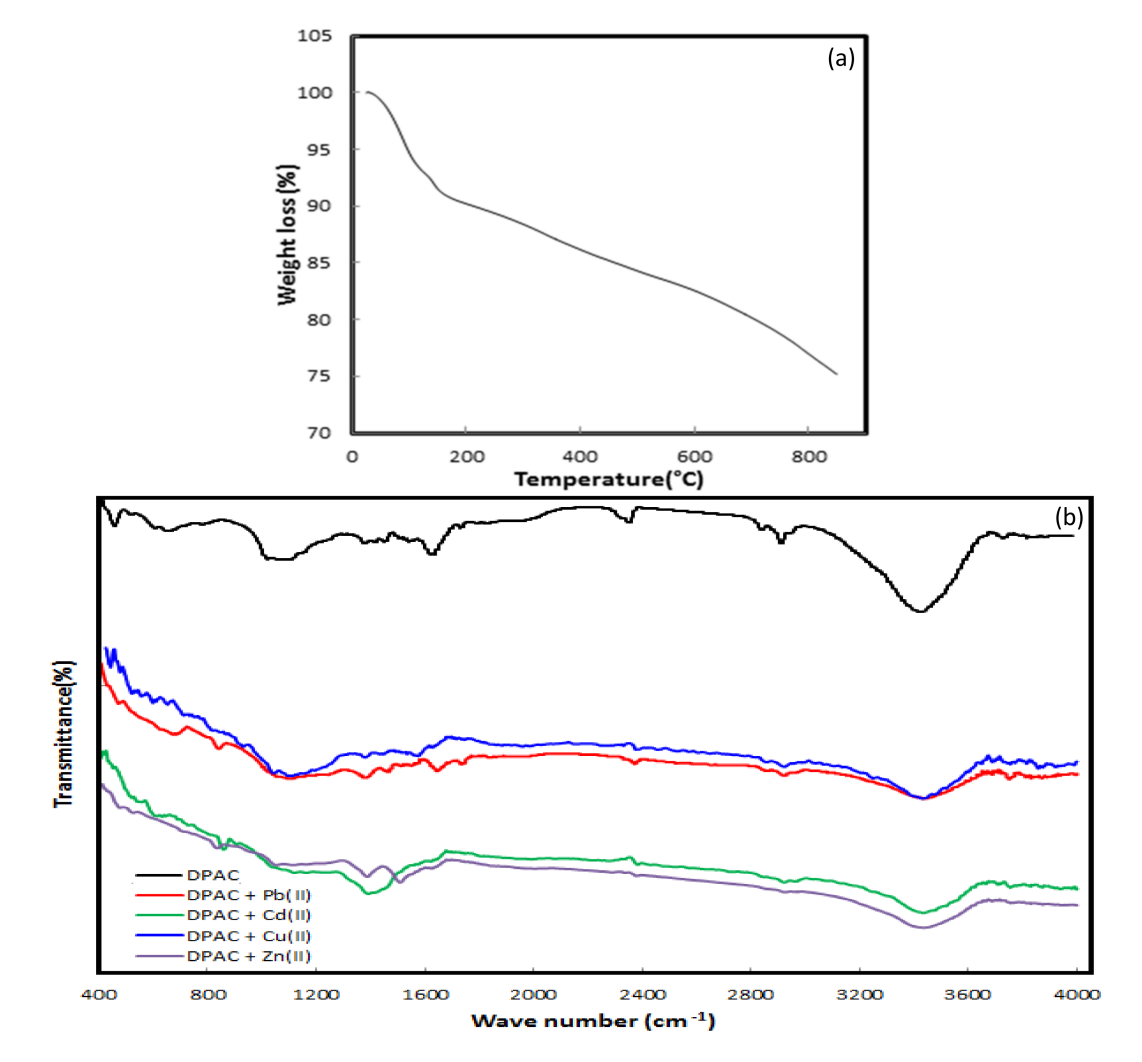
TGA plot (a), FT-IR spectra of DPAC before and after heavy metals adsorption (b).

**Fig 5** illustrates the surface morphology of DPAC before **(a)** and after heavy metals **(b–e)** adsorption. A highly porous surface with non-uniform pores of varied size for virgin DPAC was observed. The surface was mesoporous, with BET surface area: 377.6 m^2^/g [24]. After heavy metals adsorption, the DPAC pores were completely occupied leading to a formation of rough and non-uniform DPAC surface having heavy metals deposited as aggregates making multi-layer heavy metals covering over DPAC surface. These morphological changes confirmed heavy metals binding over DPAC surface. **S1 Table** presents the elemental composition of DPAC (before and after heavy metals adsorption). Before heavy metals adsorption, C, O, and traces of Na were present on DPAC surface. The presence of Na in traces confirmed successful activation of DPAC with NaOH. A little decrease in C content and an appearance of heavy metals traces on DPAC surface were observed, while Na traces were disappeared after heavy metals adsorption. This showed that ion-exchange might be a possible heavy metals adsorption mechanism on DPAC. The thermal stability of DPAC was tested by thermogravimetric (TGA) analysis in temperature range: 25–850°C. **Fig 6A** showed the occurrence 28.3% total weight loss in two-stages for aforementioned temperature range. A first stage weight loss for temperature range: ~ 25–165°C was 8.3%, occurred due to a loss volatile gases and moisture. During second stage, 20% DPAC weight loss for temperature range: ~ 165–800°C was observed due to the decomposition of hemicellulose, cellulose and lignin to tar and gases. **Fig 6B** presents the FT-IR spectra of DPAC before and after heavy metals adsorption. Results showed the presence of hydroxyl and carboxyl groups over DPAC surface. A strong absorption band characteristic of O–H stretching vibrations in DPAC appeared at 3420 cm^-1^. A strong bands at 2920 and 2825 cm^-1^ corresponds to alkane C–H stretching vibrations. The peaks at 1720 and 1600 cm^-1^ corresponds to the stretching of C = O and C = C, respectively. A peak between 1410 and 1370 cm^-1^ corresponds to C–H bending and rocking in alkane, respectively. Additionally, the DPAC spectrum showed absorption peaks at 1140.53 and 1060 cm^-1^ which characterize the C-O group. A slight spectral shift or decrease/disappearance in peaks positions due to metal binding on DPAC surface was observed after heavy metals adsorption, attributed to changes in counter ions associated with carboxylate and hydyroxylate anions, suggesting that acidic groups, carboxyl and hydroxyl, were predominant contributors in metal ions uptake. [42] Moreover, the formation of new peaks at 820, 825, 845, and 885 cm^-1^attributed to Zn–O, Pb–O, Cd–O, and Cu–O, respectively were appeared in DPAC spectra after heavy metals adsorption. Similar spectral peaks for metal oxides were appeared elsewhere. [43].

## Conclusions

Mesoporous mercerized date pit activated carbon (DPAC) having extremely high BET surface area showed promising results for the adsorption of Cd(II), Pb(II), Cu(II), and Zn(II) from aqueous medium. Preliminary adsorption studies showed maximum adsorption of Cd(II) onto DPAC followed by Cu(II) > Pb(II) > Zn(II). The experimental parameters have a profound influence on heavy metals adsorption process. Physical adsorption process having exothermic nature was observed. In general, the adsorption kinetics was rapid for studied *C*_*o*_ range. The maximum amounts of heavy metals were eluted with 0.1 M HCl. A two-step 28.3% DPAC sample total weight loss in temperature range: 25–850°C was observed during thermal analysis. A change/disappearance of peaks associated with acid functionalities during infra-red spectral analysis confirmed their involvement in heavy metals adsorption on DPAC.

## Supporting information

S1 Fig Heavy metals adsorption in single metal system on DPAC. (DOCX)

S1 Table Elemental analysis data. (DOCX)

S1 Text Adsorption isotherm models. (DOCX)

S2 Text Adsorption kinetics models. (DOCX)
