# Supplementary material for: Mercerized mesoporous date pit activated carbon—A novel adsorbent to sequester potentially toxic divalent heavy metals from water
Source: PLoS One. 2017 Sep 14;12(9):e0184493. doi: 10.1371/journal.pone.0184493 (PMC5598982; doi:10.1371/journal.pone.0184493)
Supplement: S1 Fig — (DOCX) [file pone.0184493.s001.docx]

**Supplementary material**

**Mercerized mesoporous date pit activated carbon – a novel adsorbent to sequester potentially toxic divalent heavy metals from water**

Abdullah Aldawsari^1^, Moonis Ali Khan^1,^*, B.H. Hameed^2^, Ayoub Abdullah Alqadami^1^, Masoom Raza Siddiqui^1^, Zeid Abdullah AlOthman^1^, A. Yacine Badjah Hadj Ahmed^1^

^1^Department of Chemistry, College of Science, King Saud University, P.O. Box 2455, Riyadh 11451, Saudi Arabia.

^2^School of Chemical Engineering, Engineering Campus, Universiti Sains Malaysia, 14300 Nibong Tebal, Penang, Malaysia

*Corresponding author’s E-mail address: [moonisalikhan@gmail.com](mailto:moonisalikhan@gmail.com); mokhan@ksu.edu.sa (M.A. Khan)

**Figure S1.** Heavy metals adsorption in single metal system on DPAC (**Experimental conditions:** m: 0.05 g, t: 24 hr, *C_o_*: 50 mg/L, V: 50 mL, agitation speed: 100 rpm, T: 298 K)
