# Supplementary material for: Mercerized mesoporous date pit activated carbon—A novel adsorbent to sequester potentially toxic divalent heavy metals from water
Source: PLoS One. 2017 Sep 14;12(9):e0184493. doi: 10.1371/journal.pone.0184493 (PMC5598982; doi:10.1371/journal.pone.0184493)
Supplement: S1 Table — (DOCX) [file pone.0184493.s002.docx]

**Supplementary material**

**Mercerized mesoporous date pit activated carbon – a novel adsorbent to sequester potentially toxic divalent heavy metals from water**

Abdullah Aldawsari^1^, Moonis Ali Khan^1,^*, B.H. Hameed^2^, Ayoub Abdullah Alqadami^1^, Masoom Raza Siddiqui^1^, Zeid Abdullah AlOthman^1^, A. Yacine Badjah Hadj Ahmed^1^

^1^Department of Chemistry, College of Science, King Saud University, P.O. Box 2455, Riyadh 11451, Saudi Arabia.

^2^School of Chemical Engineering, Engineering Campus, Universiti Sains Malaysia, 14300 Nibong Tebal, Penang, Malaysia

*Corresponding author’s E-mail address: [moonisalikhan@gmail.com](mailto:moonisalikhan@gmail.com); mokhan@ksu.edu.sa (M.A. Khan)

**Table S1.** Elemental analysis data

| **Sample** | **Elemental content (At. %)** | | | | | | |
| --- | --- | --- | --- | --- | --- | --- | --- |
|  | C | O | Cu | Pb | Zn | Cd | Na |
| DPAC | 94.75 | 4.60 | - | - | - | - | 0.39 |
| DPAC + Pb(II) | 89.71 | 9.86 | - | 0.43 | - | - | - |
| DPAC + Cd(II) | 85.46 | 11.58 | - | - | - | 2.96 | - |
| DPAC + Cu(II) | 87.77 | 8.66 | 3.57 | - | - | - | - |
| DPAC + Zn(II) | 83.35 | 14.04 | - | - | 2.60 |  |  |
