# Supplementary material for: Mercerized mesoporous date pit activated carbon—A novel adsorbent to sequester potentially toxic divalent heavy metals from water
Source: PLoS One. 2017 Sep 14;12(9):e0184493. doi: 10.1371/journal.pone.0184493 (PMC5598982; doi:10.1371/journal.pone.0184493)
Supplement: S1 Text — (DOCX) [file pone.0184493.s003.docx]

**Supplementary material**

**Mercerized mesoporous date pit activated carbon – a novel adsorbent to sequester potentially toxic divalent heavy metals from water**

Abdullah Aldawsari^1^, Moonis Ali Khan^1,^*, B.H. Hameed^2^, Ayoub Abdullah Alqadami^1^, Masoom Raza Siddiqui^1^, Zeid Abdullah AlOthman^1^, A. Yacine Badjah Hadj Ahmed^1^

^1^Department of Chemistry, College of Science, King Saud University, P.O. Box 2455, Riyadh 11451, Saudi Arabia.

^2^School of Chemical Engineering, Engineering Campus, Universiti Sains Malaysia, 14300 Nibong Tebal, Penang, Malaysia

*Corresponding author’s E-mail address: [moonisalikhan@gmail.com](mailto:moonisalikhan@gmail.com); mokhan@ksu.edu.sa (M.A. Khan)

**Text S1. Adsorption isotherm models**

Langmuir isotherm model assumes formation of monomolecular layer over adsorbent surface without interaction between the adsorbed molecules. Langmuir isotherm model in non-linearized and linearized form is expressed as:^1^

$$q_{e}=\frac{q_{m}bC_{e}}{1+bC_{e}} (S1)$$

$$\frac{C_{e}}{q_{e}}=\frac{1}{bq_{m}}+\frac{1}{q_{m}}\times C_{e} (S2)$$

where *q_m_* (mg/g) and *b* (L/mg) are the constants for maximum monolayer adsorption capacity and a constant related to the heat of adsorption, respectively.

The essential feature of Langmuir isotherm can be expressed by separation factor (*R_L_*), a dimensionless constant, can be represented as:

$$R_{L}=\frac{1}{1+bC_{o}} (S3)$$

The magnitude of *R_L_* reflects the nature of adsorption. If *R_L_* > 1(unfavorable adsorption process), 0 < *R_L_* < 1 (favorable adsorption process), *R_L_* =1 (linear adsorption process), *R_L_* = 0 (irreversible adsorption process).

Freundlich isotherm in linear and non-linear form is expressed as: ^2^

$q_{e}=K_{f}C_{e}^{\frac{1}{n}}$ (S4)

$$logq_{e}=log K_{f}+\frac{1}{n} logC_{e} (S5)$$

where *K_f_* ((mg/g) (L/mg)^(1/n)^) and *n* are the Freundlich constants related to bonding energy and deviation in adsorption from linearity, respectively. If *n =*1 (linear adsorption process), *n* < 1 (chemical adsorption process), *n* > 1 (physical adsorption process).

**References**

(1) [I.](http://pubs.acs.org/author/Langmuir%2C+Irving) Langmuir, J. Am. Chem. Soc., 1918, 40, 1361.

(2) H.M.F. Freundlich,  J. Phys. Chem., 1906, 57, 385.
