# Supplementary material for: Mercerized mesoporous date pit activated carbon—A novel adsorbent to sequester potentially toxic divalent heavy metals from water
Source: PLoS One. 2017 Sep 14;12(9):e0184493. doi: 10.1371/journal.pone.0184493 (PMC5598982; doi:10.1371/journal.pone.0184493)
Supplement: S2 Text — (DOCX) [file pone.0184493.s004.docx]

**Supplementary material**

**Mercerized mesoporous date pit activated carbon – a novel adsorbent to sequester potentially toxic divalent heavy metals from water**

Abdullah Aldawsari^1^, Moonis Ali Khan^1,^*, B.H. Hameed^2^, Ayoub Abdullah Alqadami^1^, Masoom Raza Siddiqui^1^, Zeid Abdullah AlOthman^1^, A. Yacine Badjah Hadj Ahmed^1^

^1^Department of Chemistry, College of Science, King Saud University, P.O. Box 2455, Riyadh 11451, Saudi Arabia.

^2^School of Chemical Engineering, Engineering Campus, Universiti Sains Malaysia, 14300 Nibong Tebal, Penang, Malaysia

*Corresponding author’s E-mail address: [moonisalikhan@gmail.com](mailto:moonisalikhan@gmail.com); mokhan@ksu.edu.sa (M.A. Khan)

**Text S2. Adsorption kinetics models**

The pseudo-first-order^1^ and pseudo-second-order^2^ kinetics models in linearized forms are expressed as:

$$log{(q}_{e}-q_{t})=logq_{e}-\frac{k_{1}}{2.303}\times t (S1)$$

$$\frac{t}{q_{t}}= \frac{1}{k_{2}q_{e}^{2}}+\frac{1}{q_{e}}\times t (S2)$$

where *q_e_* and *q_t_* are the adsorption capacities at equilibrium and at time *t*, respectively, *k_1_* and *k_2_* are the pseudo-first-order and pseudo-second-order rate constants, respectively.

**References**

(1) S. Lagergren, *Kung. Sven. Vetens. Hand*., 1898, 24, 1.

(2) Ho, Y.S.; McKay, G., *Water Res.*, 2000, 34, 735.
